# Tobacco smoke exposure and the risk of childhood acute lymphoblastic leukemia and acute myeloid leukemia: A meta-analysis

**DOI:** 10.1097/MD.0000000000016454

**Published:** 2019-07-12

**Authors:** Dong Chunxia, Wang Meifang, Zhang Jianhua, Zhang Ruijuan, Liu Xiue, Zheng Zhuanzhen, Yang Linhua

**Affiliations:** Department of Hematology, The Second Hospital of Shanxi Medical University, Taiyuan, Shanxi, China.

**Keywords:** childhood acute lymphoblastic leukemia/acute myeloid leukemia, tobacco smoke exposure

## Abstract

**Objective:**

Tobacco smoke contains carcinogens known to damage somatic and germ cells. In this study, we investigated the effect of tobacco smoking on the risk of childhood acute lymphoblastic leukemia (ALL) and myeloid leukemia (AML).

**Methods:**

Information about tobacco smoking exposures of the mother before, during, and after pregnancy was collected via PubMed, Embase, and Web of Science databases through November 5, 2018. We performed to evaluate the association between smoking exposure and the risk of childhood ALL and AML. Study selection, data abstraction, and quality assessment were performed by 2 independent reviewers. Random effects models were used to obtain summary odds ratios (ORs) and 95% confidence intervals (CIs).

**Results:**

Nineteen case–control studies of childhood leukemia (age < 15 years) conducted in 9 countries from 1974 to 2018. Maternal smoking exposures did not a significant association with childhood ALL (OR = 1.004, 95% CI 0.953–1.058, *P* = .881) and AML (OR = 0.92, 95% CI 0.815–1.038, *P* = .177) during exposure time windows. However, there was an association with paternal smoking and ALL (OR = 1.15, 95% CI 1.038–1.275, *P* = .007). Paternal smoking in AML showed there was no association with smoking exposures and childhood AML (OR = 1.133, 95% CI 0.943–1.362, *P* = .181). Next, maternal daily cigarettes consumption showed no associations with ALL (OR = 1.08, 95% CI 1.000–1.168, *P* = .051) during pregnancy. No association with maternal daily smoking and AML (OR = 0.909, 95% CI 0.682–1.211, *P* = .514). Paternal daily cigarettes consumption was associated with increased risks of childhood ALL (OR = 1.200, 95% CI 1.112–1.302, *P* = .000). The higher consumption of paternal smoking (more than 10 per day) was significantly related to childhood ALL. Paternal daily smoking consumption also was related to AML (OR = 1.242, 95% CI 1.031–1.496, *P* = .022).

**Conclusion:**

Maternal smoking before, during, or after pregnancy was not associated with childhood ALL or AML. However, paternal smoking was related to a significantly elevated risk of childhood ALL during pregnancy, but not for AML. Maternal daily smoking consumption was not associated with ALL or AML during pregnancy. The higher consumption of paternal smoking were, the higher the risk of childhood ALL or AML.

## 1 Introduction

Acute leukemia is the most common childhood cancer, acute lymphoblastic leukemia (ALL) accounts for 75% to 80% of total cases of childhood leukemia, acute nonlymphocytic leukemia (AnLL) for about 20%.^[1]^ Many studies have examined the potential precipitating factors of acute leukemia. For instance, benzene is known to damage cells of myeloid lineage and pluripotent hematopoietic stem cells,^[2]^ which potentially playing a role in the development of both childhood ALL and acute myeloid leukemia (AML). Meanwhile, there also are other risk factors including: car exhaust fumes, pesticides, antiepileptic drugs, chemical contamination in drinking water, both viral and bacterial infections, and parental cigarette smoking, and so on.^[3]^ Many studies have proven carcinogens are present in tobacco,^[4]^ which is known to increase the risk of various adult cancers.^[5]^ The cause lies in smoking is associated with oxidative damage and aneuploidy of sperm.^[6]^ Tobacco smoke has increased frequencies of chromosomal abnormalities.^[7]^ Tobacco-related contaminants can damage DNA in human somatic cells, and there is growing evidence that tobacco affects germ cells not only in animals, but in humans.^[8]^

There are strong reasons for considering parental smoking behavior as a risk factor for childhood cancer. Many studies showed that active tobacco smoking is an established risk for adult myeloid leukemia.^[9]^ A case–control investigation of childhood ALL was conducted showed that maternal smoking was associated significantly with childhood ALL.^[10]^ However, a study from the French demonstrated there was no effect of maternal smoking on the childhood acute lymphoblastic risk.^[11]^ Although the association may be biologically plausible, it is less clear whether tobacco smoke exposure was related with acute leukemia. Many studies contradict and increase people's confusion.

We conducted a systematic review and meta-analysis to investigate risk factor between tobacco smoke exposures and childhood leukemia during critical exposure time windows (preconception, pregnancy, and childhood).

## 2 Methods

Sine this study is a meta-analysis of previously published studies, the ethical approval and patient consent are not required. This study was conducted and reported in adherence to Preferred Reporting Items for Systematic Reviews and Meta-analysis.

### 2.1 Literature search

The PubMed, Embase, and Web of Science databases were systematically searched for relevant studies until November 5, 2018. The following keywords were used individually and in combination: “acute lymphoblastic leukemia,” “acute myeloid leukemia,” and “tobacco smoke exposure.”

### 2.2 Inclusion and exclusion criteria

We choose original epidemiologic studies of childhood leukemia using a case–control study design with an assessment of smoking exposure and childhood ALL or AML. The following studies were excluded: letters, reviews, case reports, conference abstracts, or expert opinions; and articles with insufficient information on smoking exposure variables.

### 2.3 Data extraction and assessment of study quality

Two investigators independently extracted data that met our inclusion and exclusion criteria. The Newcastle–Ottawa scale was used to evaluate the methodologic quality of all included case–control studies.^[12]^

### 2.4 Statistical analysis

Pooled estimates of odds ratios (ORs) with 95% confidence intervals (CIs) were used to evaluate the associations between tobacco smoking exposures and childhood acute leukemia. To stratify the data for analysis, (maternal or paternal smoking in ALL/AML, paternal or maternal daily cigarettes in ALL/AML). Based on the Chi-squared statistic *Q*, inter-study heterogeneity was assumed^[13]^ in cases in which *I*^2^ > 50%, and ORs were pooled according to random-effects models. Alternatively, fixed-effects models were used. All statistical analyses were performed using Stata 13.0 (Stata Corporation, College Station, TX). A *P*-value <.05 were considered statistically significant.

## 3 Results

### 3.1 Study selection and characteristics

**Figure 1:**
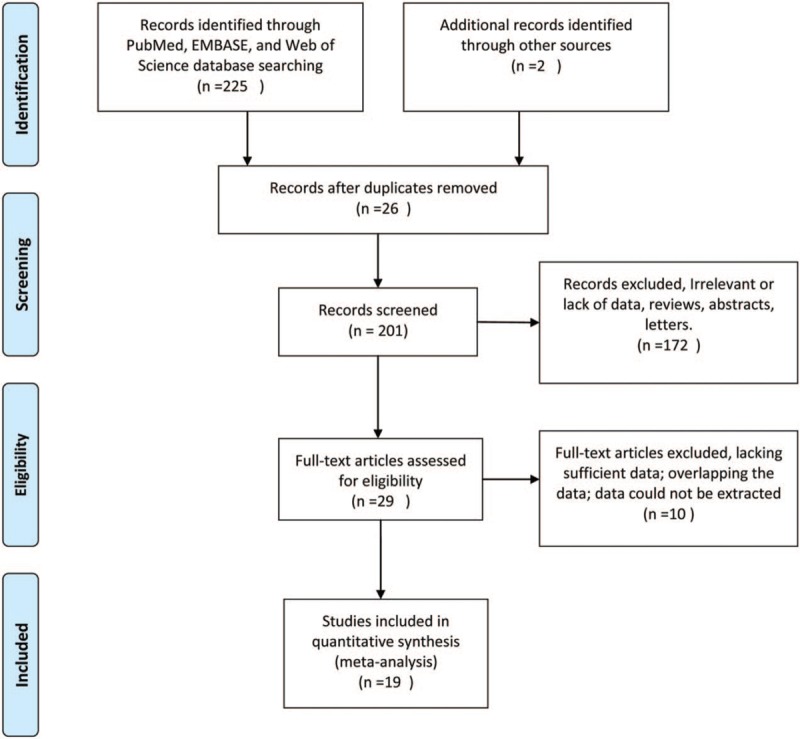
Flow chart of study selection process.

The literature search was conducted on November 5, 2018. The detailed steps of the systematic search and selection process are given as a flow diagram (Fig. 1). The searches yielded 227 potentially eligible titles. After removing duplicate articles and reviewing the abstracts, the full text of 29 articles were obtained and compared to the inclusion criteria. Ten articles were excluded (Fig. 1). This resulted in 19 eligible articles (Table 1). These were all case–control studies for smoking mother research. The main characteristics of the included studies are presented in Table 1. Nineteen case–control studies of childhood leukemia (age <15 years) conducted in 9 countries from 1974 to 2018. The following data were extracted: author's name, research year, country, case recruitment, control selection, matching, and sample sizes. Data were available for the pooled analyses.

**Table 1:**
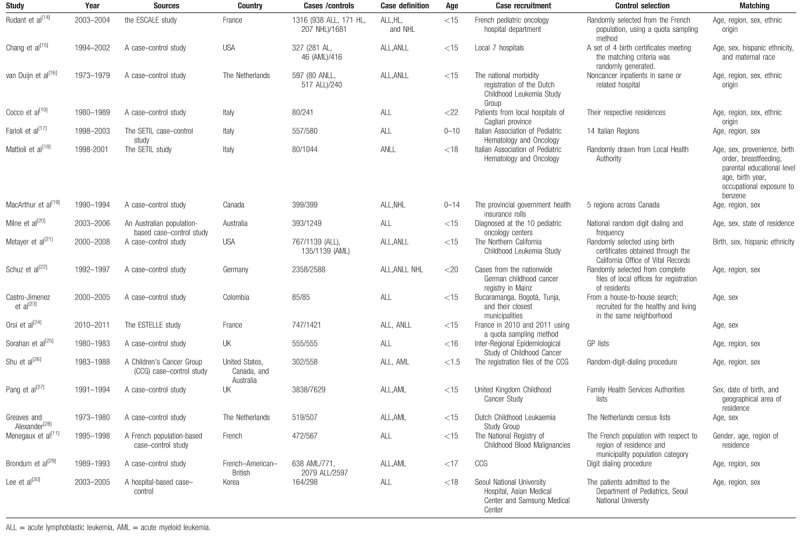
Characteristics of included studies.

### 3.2 Correlating tobacco smoke exposure with childhood acute lymphoblastic during exposure time windows (preconception, pregnancy, and postnatal)

**Figure 2:**
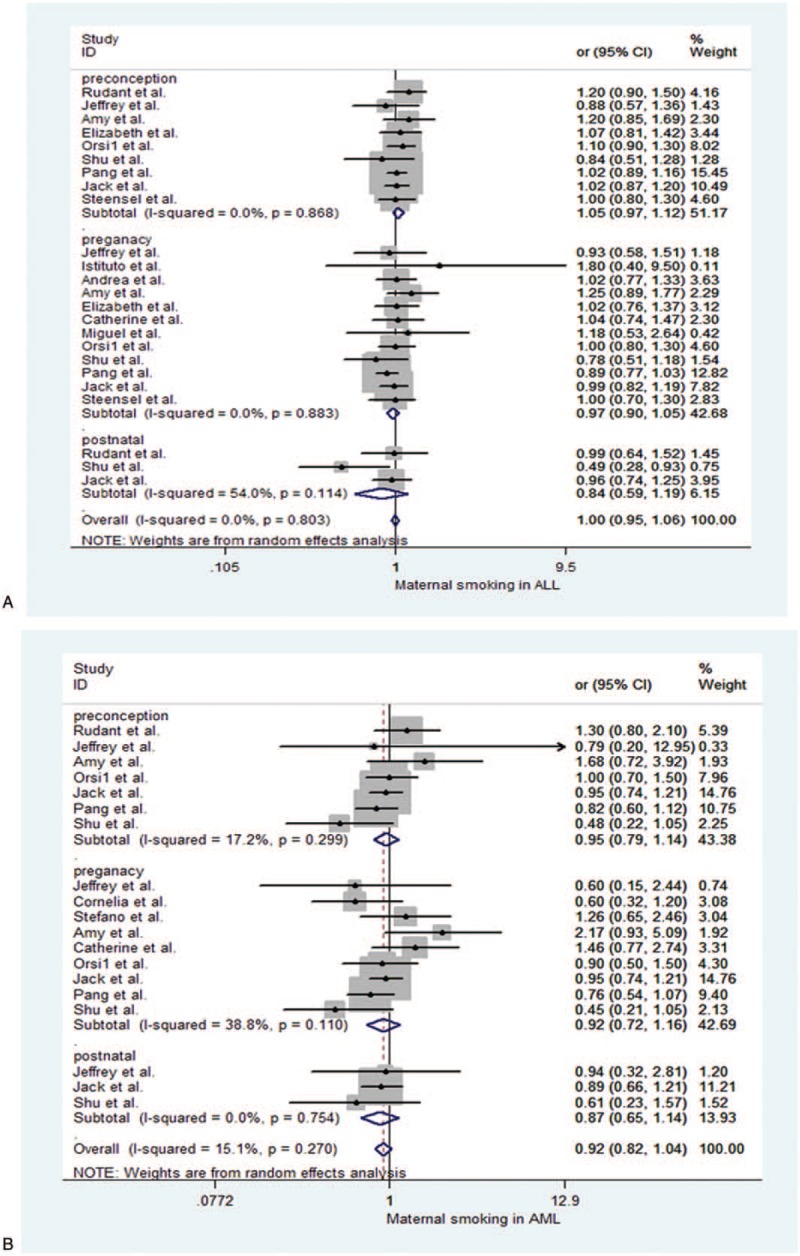
Forest plot depict parental smoking and odds ratios (ORs) for parental smoking in childhood acute lymphoblastic during exposure time windows. (A) Maternal smoking in acute lymphoblastic leukemia. (B) Maternal smoking in acute myeloid leukemia.

**Figure 3:**
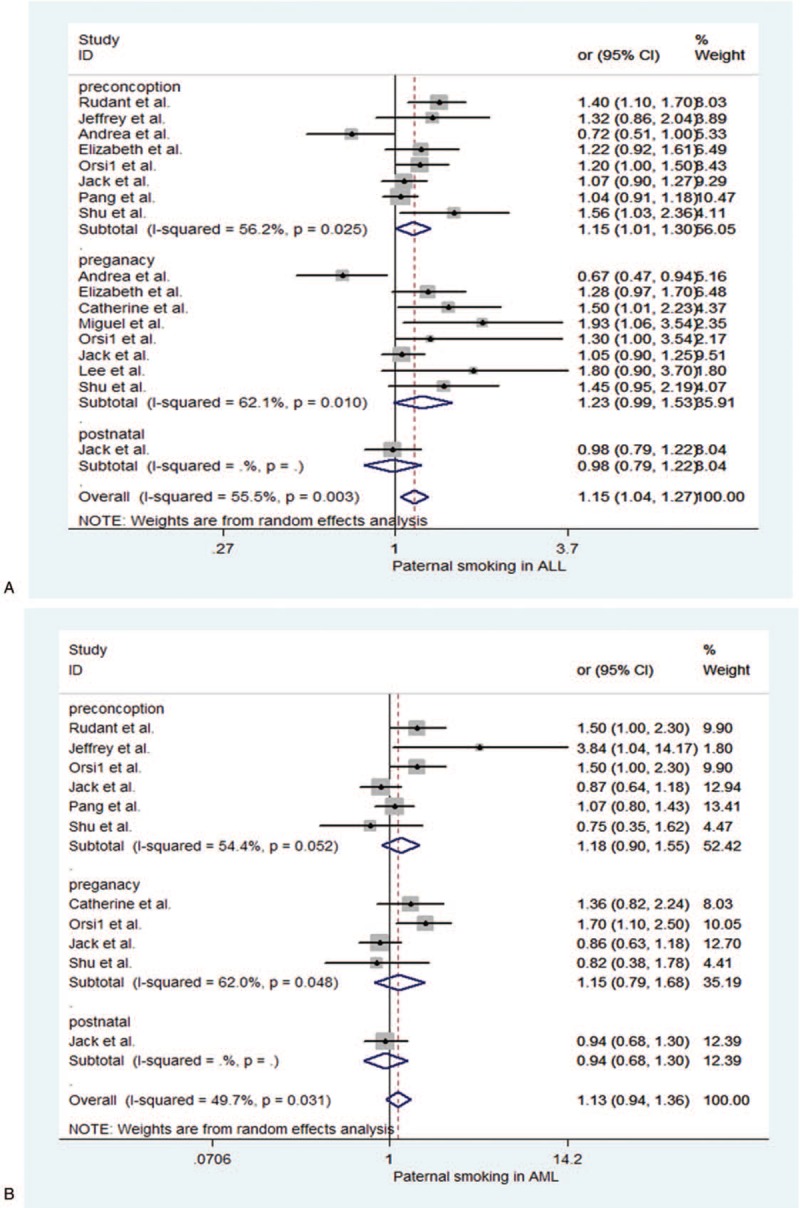
Forest plot depict parental smoking and odds ratios (ORs) for parental smoking in childhood acute lymphoblastic during exposure time windows. (A) Paternal smoking in acute myeloid leukemia (AML). (B) Paternal smoking in AML.

We examined the relationship between parental smoking exposure and childhood acute leukemia during exposure time windows (Figs. 2 and 3 and Table 2). As showed in the Figure 2A and Table 2, in childhood ALL maternal smoking, there was no significant heterogeneity was found (*I*^2^ = 0.0%, *P* = .803). Our results showed that maternal smoking exposure were not associated with childhood ALL (OR = 1.004, 95% CI 0.953–1.058, *P* = .881). In subgroups, maternal preconception smoking subgroup were OR = 1.046, 95% CI (0.972–1.125), *P* = .23. And pregnancy subgroup were OR = 0.973, 95% CI (0.898–1.054) *P* = .500, and postnatal subgroup were OR = 1.004, 95% CI (0.953–1.058), *P* = .317. In these maternal smoking subgroups, there was no evidence of a general tendency for increased the ALL risk.

**Table 2:**
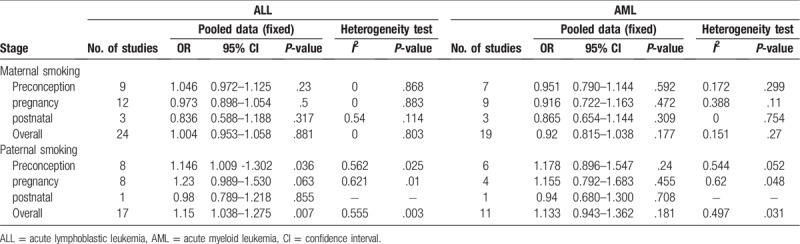
Odd risks (OR) of childhood ALL associated with tobacco smoke during exposure time windows.

When maternal smoking were in childhood AML in the Figure 2B and Table 3, our data also showed that no heterogeneity was found (*I*^2^ = 15.1%, *P* = .27). There was no significant association with maternal smoking exposures and childhood AML during exposure time windows (OR = 0.92, 95% CI 0.815–1.038, *P* = .177). At the same time, for the maternal preconception, pregnancy, and postnatal subgroup, the results were respectively (OR = 0.951, 95% CI 0.790–1.144, *P* = .592) in preconception; (OR = 0.916, 95% CI 0.722–1.163, *P* = .472) in pregnancy; and (OR = 0.865, 95% CI 0.654–1.144, *P* = .309) in postnatal subgroup. All subgroups were no significant with childhood AML.

**Table 3:**
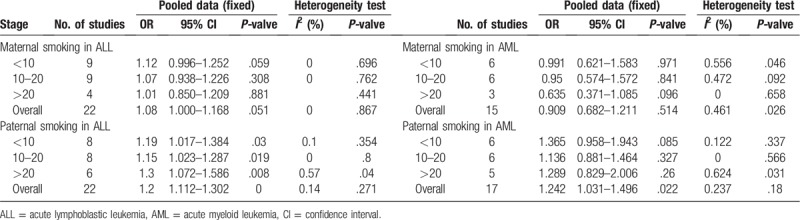
Odd risks (OR) of childhood ALL associated with parental daily consumption of cigarettes during pregnancy.

Next, paternal smoking in ALL was showed in the Figure 3A and Table 2. We found there was an association with paternal smoking and ALL (OR = 1.15, 95% CI 1.038–1.275, *P* = .007). For paternal smoking preconception subgroup was also associated with an increased ALL risk (OR = 1.146, 95% CI 1.009–1.302, *P* = .036). The result was significant. But paternal pregnancy and postnatal subgroup were, respectively, no significance: OR = 1.23, 95% CI (0.989–1.530), *P* = .063, pregnancy, OR = 0.98, 95% CI (0.789–1.218), *P* = .855, postnatal.

Paternal smoking in AML was showed in the Figure 3B and Table 2. Our results showed there was no association with paternal smoking and childhood AML (OR = 1.133, 95% CI 0.943–1.362, *P* = .181). In subgroups, the results also were no significant. They were respectively OR = 1.178, 95% CI (0.896–1.547), *P* = .240, preconception, OR = 1.155, 95% CI (0.792–1.683), *P* = .455, pregnancy, and OR = 0.940, 95% CI (0.680–1.300), *P* = .708, postnatal.

Overall, maternal smoking before, during, or after pregnancy was not associated with childhood ALL and AML. However, paternal smoking, particularly before pregnancy, was significantly related to an elevated risk of childhood ALL. But not for AML, which was no significance.

### 3.3 Childhood ALL with parental daily consumption of cigarettes during pregnancy

**Figure 4:**
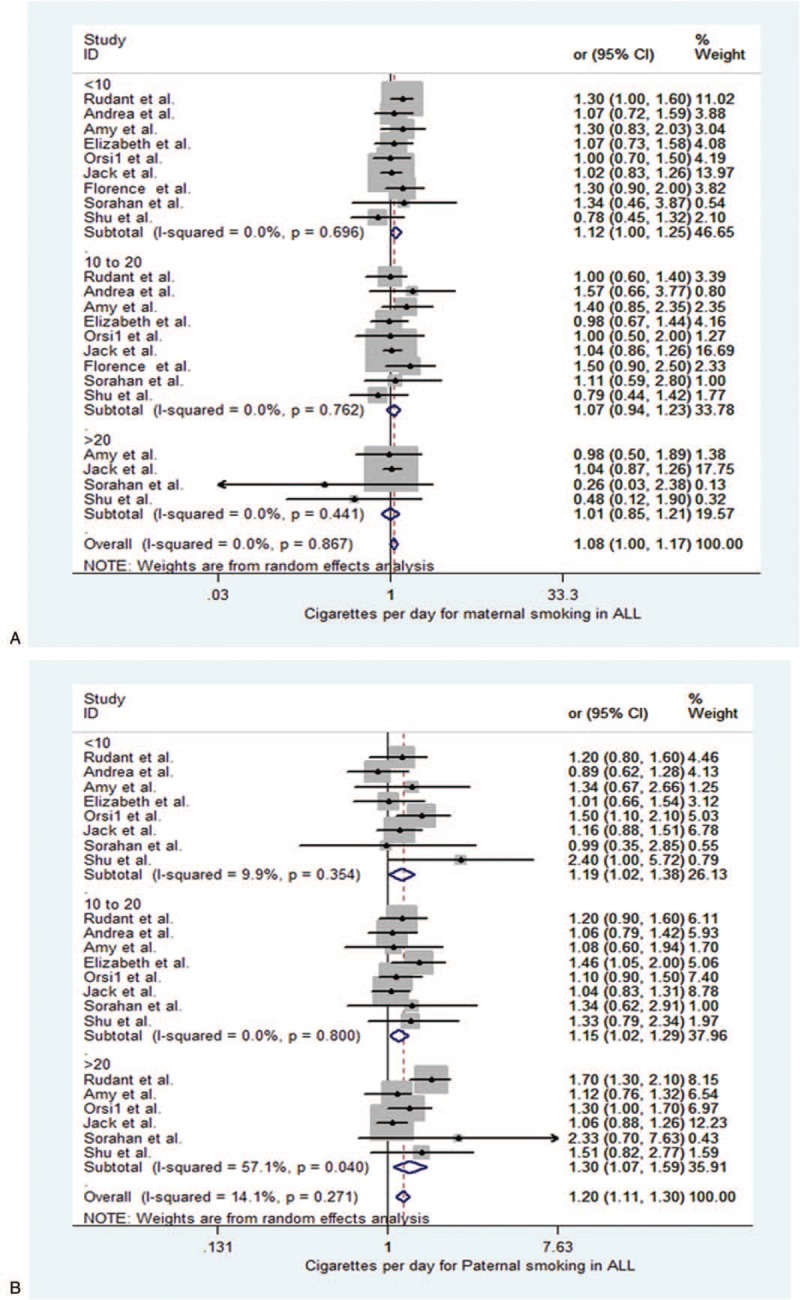
Forest plot depict childhood acute lymphoblastic leukemia (ALL) with parental cigarettes daily consumption during pregnancy. (A) Cigarettes per day for maternal smoking in ALL. (B) Cigarettes per day for paternal smoking in ALL.

As showed in the Figure 4A and Table 3 for maternal daily cigarettes consumption in ALL, which show childhood ALL have no associations with maternal daily consumption cigarettes during pregnancy (OR = 1.08, 95% CI 1.000–1.168, *P* = .051). However, there were slightly an increased risks for ALL (OR = 1.12, 95% CI 0.996–1.252, *P* = .059) in <10 maternal cigarettes consumption subgroup, but not statistically significant. While, 10 to 20 subgroup and >10 subgroup showed no associations with childhood ALL during pregnancy. Their results were OR = 1.07, 95% CI (0.938–1.226), *P* = .308, 10 to 20 cigarettes/day and OR = 1.01, 95% CI (0.850–1.209), *P* = .881, >20 cigarettes/day). Both were not associated with increased risks of childhood ALL in pooled analyses.

Paternal daily cigarette consumption in ALL was showed in the Figure 4B and Table 3. The paternal daily cigarette consumption was associated with the increased risks of childhood ALL (OR = 1.200, 95% CI 1.112–1.302, *P* = .000). In subgroups, paternal smoking <10 subgroup showed an associations with childhood ALL during pregnancy (OR = 1.190, 95% CI 1.017–1.384, *P* = .030, <10 subgroup). Moreover, the higher consumption of paternal smoking (more than 10 per day) was also significantly related to childhood ALL. Paternal smoking 10 to 20 cigarettes per day was OR = 1.150, 95% CI (1.023–1.287), *P* = .019; >20 was OR = 1.300, 95% CI (1.072–1.586), *P* = .008, during pregnancy. Both were statistically significant increased risks of childhood ALL for father smoking during pregnancy.

**Figure 5:**
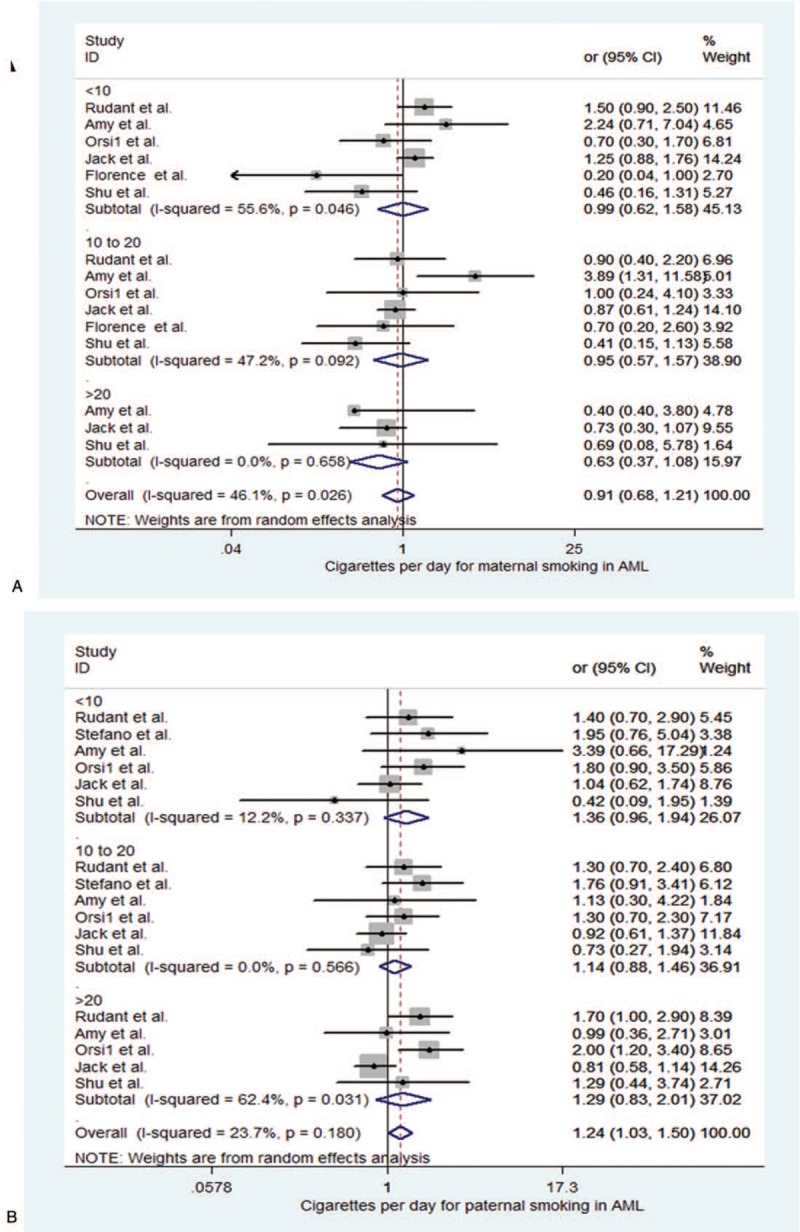
Forest plot depict childhood acute lymphoblastic leukemia with parental cigarettes daily consumption during pregnancy. (A) Cigarettes per day for maternal smoking in acute myeloid leukemia (AML). (B) Cigarettes per day for paternal smoking in AML.

As showed in the Figure 5A and Table 3 for maternal cigarettes daily consumption in AML during pregnancy. No association with maternal cigarettes consumption per day and AML was found (OR = 0.909, 95% CI 0.682–1.211, *P* = .514). In the subgroups, neither <10, 10 to 20, or >20 was association with AML. The results were, respectively, OR = 0.991, 95% CI (0.621–1.583), *P* = .971, <10 cigarettes per day; cigarettes per day OR = 0.95, 95% CI (0.574–1.572), *P* = .841, 10 to 20 cigarettes per day; and OR = 0.635, 95% CI (0.371–1.085), *P* = .096, >20 cigarettes per day. Meanwhile, there also were no heterogeneity (*I*^2^ = 46.1%, 0.026).

Interestingly, as showed in the Figure 5B and Table 3, we found that paternal cigarettes consumption daily was related to childhood AML (OR = 1.242, 95%CI 1.031–1.496, *P* = .022). In the subgroups, paternal cigarettes consumption <10 per day during pregnancy, there was a slightly trend for the risk of childhood AML (OR = 1.365, 95% CI 0.958–1.943, *P* = .085), but not statistically significant. Moreover, 10 to 20 or >20 subgroup was no association with childhood AML. The results were respectively 10 to 20 subgroup (OR = 1.136, 95% CI 0.881–1.464, *P* = .327), >20 cigarettes per day (OR = 1.289, 95% CI 0.829–2.006, *P* = .260). Meanwhile, there were no heterogeneity (*I*^2^ = 23.7%, *P* = .18).

Overall, the interaction between maternal daily cigarettes consumption during pregnancy and childhood ALL, AML was not significant. While a significant association between paternal cigarettes consumption and childhood ALL, AML was observed during pregnancy (Table 3).

**Figure 6:**
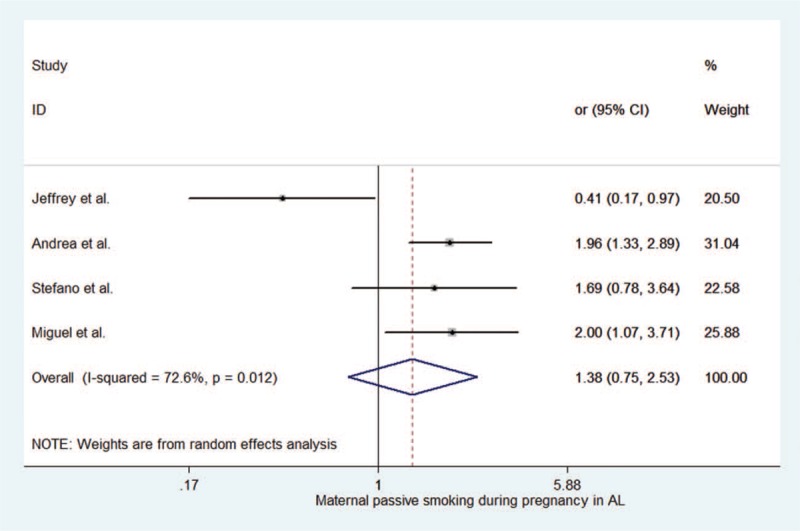
Forest plot depiction of maternal passive smoking during pregnancy in acute lymphoblastic leukemia.

When maternal passive smoking during pregnancy in ALL (Fig. 6 and Table 4), our results were (summary OR = 1.383, 95% CI 0.755–2.533). The *P*-value was .294. It showed maternal passive smoking was no associated with an increased risk of childhood ALL.

**Table 4:**
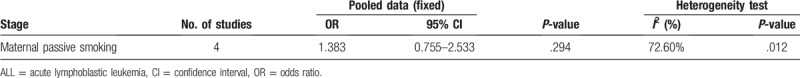
Maternal passive smoking during pregnancy in childhood ALL.

## 4 Discussion

Previous studies suggested that tumor in children may be caused by noxious substance exposures early in life.^[31]^ Tobacco contains several mutagenic and carcinogenic compounds to cause human germ cell mutations during spermatogenesis.^[10,32]^ Some studies displayed smoking can have adverse effects on the health of the baby during pregnancy.^[33,34]^ Several lines of evidence support the potential role of tobacco in the pathogenesis of cancer.^[35]^ In this study, we investigate the relationship of parental smoking exposure and the risk of ALL and AML during exposure time windows (preconception, pregnancy, postnatal).

Previous case–control studies have tended to show weak associations between maternal smoking and childhood ALL.^[20,36,37]^ A study from the French demonstrated no effect of maternal smoking during the index pregnancy on the childhood acute lymphoblastic risk.^[11]^ Greaves and Alexander also showed no association between childhood leukemia and maternal smoking exposure.^[28]^ Milne et al demonstrated that maternal smoking was not increased the risk of childhood ALL.^[20]^ Our data showed that maternal smoking before, during, or after pregnancy was not associated with childhood ALL.

Several studies examined the potential association between childhood leukemia and tobacco. Active maternal smoking during pregnancy has been associated with a higher risk of behavior disorders in children.^[34]^ An United Kingdom Childhood Cancer Study showed that a statistically significant increased risk of developing hepatoblastoma was found in children whose mothers smoked preconceptionally (OR = 2.68, *P* = .02) and strongest for both parents smoking (OR = 4.74, *P* = .003).^[27]^ Our findings suggested only maternal preconception subgroups was a slightly trend with childhood ALL.

In AML, a study showed that maternal smoking during pregnancy was negatively associated with infant leukemia AML risk (OR = 0.45, 95% CI = 0.21–0.96).^[26]^ Brondum et al showed that maternal smoking was no significant risk of AML (OR = 0.95, 95% CI 0.74–1.22).^[29]^ Our data showed that maternal smoking before, during, or after pregnancy was not associated with childhood AML, by which did not elevate risk for either AML.

Next, Menegaux et al demonstrated that no association with parental smoking, either maternal or paternal, was observed with ALL.^[11]^ However, a national registry-based case–control study ESCALE was carried out in France, which was paternal smoking significantly associated with childhood ALL (OR = 1.4, 95% CI 1.1–1.7), AML (OR = 1.5, 95% CI 1.0–2.3),^[14]^ by which it was related to an elevated risk of ALL.^[27]^ Our results showed that paternal smoking was related to a significantly elevated risk of childhood ALL, but for AML, in which was no significance.

Interestingly, Milne et al demonstrated that the OR for paternal smoking of 15 cigarettes per day around the time of the child's conception was 1.35 (95% CI 0.98–1.86).^[20]^ Orsi et al study showed that preconception paternal daily smoking consumption was significantly associated with ALL (OR = 1.2, CI 1.1–1.5) and AML (OR = 1.5, CI 1.0–2.3).^[24]^ Our data showed that paternal daily smoking consumption during pregnancy was observed a significant association with ALL and AML.

An Australian Study of Causes of ALL study showed that a heavier paternal smoking around the time of conception is a risk factor for childhood ALL (OR = 1.15, 95% CI 1.06–1.24) for any paternal smoking around the time of the child's conception and for smoking 20 cigarettes per day at that time (OR = 1.44, 95% CI 1.24–1.68).^[20]^ Results were broadly in line with those of previous studies. Our analysis did supported a productive evidence that the higher paternal tobacco daily consumption, the higher was the risk of childhood ALL. A heavier smoking does appear to increase this risk. At the same time, the high correlation paternal daily smoking consumption was related with ALL between pregnancy and postnatal paternal smoking. Our findings indicate that both the timing and dose of paternal smoking are important in influencing risk of childhood ALL and AML.

Consistent with most previous studies,^[38]^ there were no association between maternal daily smoking consumption and risk of childhood ALL or AML during pregnancy. Maternal daily smoking consumption during pregnancy was negatively associated with childhood AML or ALL. Meanwhile, in most past studies on passive smoke exposure of children and risk of AnLL, most findings were negative.^[39]^ Our results for maternal passive smoke also suggested a negative association with ALL.

Our study also have several limitations: the present study is more likely to be subject to reporting bias because of increased public awareness of adverse effects of smoking and blinding parents with respect to the study hypothesis regarding smoking was impracticable. These data were collected at a time when there was little pressure on mothers to stop smoking during pregnancy and therefore less liability to bias. More importantly, since smoking is such a well-known risk factor for cancer and for unfavorable pregnancy outcomes, the possibility of guilt feelings leading to underreporting, especially in case mother, must be considered. Next, childhood leukemia is a heterogeneous disease and epidemiologic studies of childhood leukemia can be greatly improved by grouping childhood leukemia into more homogeneous groups by molecular techniques and assess gene–environment interaction.

## 5 Conclusion

Our study supports that paternal smoking is associated with the risk of childhood ALL and AML during pregnancy, but not for maternal smoking. Further studies are needed to confirm the association of paternal smoking with increased risk of childhood ALL in offspring.

## Author contributions

**Conceptualization:** Dong Chunxia, Wang Meifang, Liu Xiue.

**Data curation:** Dong Chunxia, Wang Meifang, Zhang Ruijuan, Liu Xiue.

**Formal analysis:** Dong Chunxia, Zhang Ruijuan, Zheng Zhuanzhen.

**Funding acquisition:** Dong Chunxia, Zheng Zhuanzhen.

**Investigation:** Dong Chunxia, Zhang Jianhua, Liu Xiue.

**Methodology:** Dong Chunxia, Zhang Jianhua, Zhang Ruijuan, Liu Xiue.

**Resources:** Dong Chunxia, Zhang Jianhua.

**Software:** Dong Chunxia, Zhang Jianhua, Zhang Ruijuan, Zheng Zhuanzhen, Yang Linhua.

**Supervision:** Zhang Ruijuan.

**Visualization:** Yang Linhua.

**Writing – original draft:** Dong Chunxia, Yang Linhua.

**Writing – review & editing:** Dong Chunxia, Yang Linhua.

## References

[1] Mezei G Sudan M Izraeli S Epidemiology of childhood leukemia in the presence and absence of Down syndrome. Cancer Epidemiol 2014;38:479–89. 25113940 10.1016/j.canep.2014.07.006

[2] Goldstein BD Benzene as a cause of lymphoproliferative disorders. Chem Biol Interact 2010;184:147–50. 20035727 10.1016/j.cbi.2009.12.021

[3] Belson M Kingsley B Holmes A Risk factors for acute leukemia in children: a review. Environ Health Perspect 2007;115:138–45. 17366834 10.1289/ehp.9023 PMC1817663

[4] Hoffmann D Djordjevic MV Hoffmann I The changing cigarette. Prev Med 1997;26:427–34. 9245661 10.1006/pmed.1997.0183

[5] IARC Working Group on the Evaluation of Carcinogenic Risks to Humans. Tobacco smoke and involuntary smoking. IARC Monogr Eval Carcinog Risks Hum 2004;83:1–438. 15285078 PMC4781536

[6] Fraga CG Motchnik PA Wyrobek AJ Smoking and low antioxidant levels increase oxidative damage to sperm DNA. Mutat Res 1996;351:199–203. 8622715 10.1016/0027-5107(95)00251-0

[7] Pluth JM Ramsey MJ Tucker JD Role of maternal exposures and newborn genotypes on newborn chromosome aberration frequencies. Mutat Res 2000;465:101–11. 10708975 10.1016/s1383-5718(99)00217-x

[8] DeMarini DM Genotoxicity of tobacco smoke and tobacco smoke condensate: a review. Mutat Res 2004;567:447–74. 15572290 10.1016/j.mrrev.2004.02.001

[9] IARC Working Group on the Evaluation of Carcinogenic Risks to Humans. Personal habits and indoor combustions. Volume 100 E. A review of human carcinogens. IARC Monogr Eval Carcinog Risks Hum 2012;100:1–538. PMC4781577 23193840

[10] Cocco P Rapallo M Targhetta R Analysis of risk factors in a cluster of childhood acute lymphoblastic leukemia. Arch Environ Health 1996;51:242–4. 8687246 10.1080/00039896.1996.9936022

[11] Menegaux F Ripert M Hemon D Maternal alcohol and coffee drinking, parental smoking and childhood leukaemia: a French population-based case-control study. Paediatr Perinat Epidemiol 2007;21:293–9. 17564585 10.1111/j.1365-3016.2007.00824.x

[12] Stang A Critical evaluation of the Newcastle-Ottawa scale for the assessment of the quality of nonrandomized studies in meta-analyses. Eur J Epidemiol 2010;25:603–5. 20652370 10.1007/s10654-010-9491-z

[13] DerSimonian R Laird N Meta-analysis in clinical trials. Control Clin Trials 1986;7:177–88. 3802833 10.1016/0197-2456(86)90046-2

[14] Rudant J Menegaux F Leverger G Childhood hematopoietic malignancies and parental use of tobacco and alcohol: the ESCALE study (SFCE). Cancer Causes Control 2008;19:1277–90. 18618277 10.1007/s10552-008-9199-5

[15] Chang JS Selvin S Metayer C Parental smoking and the risk of childhood leukemia. Am J Epidemiol 2006;163:1091–100. 16597704 10.1093/aje/kwj143

[16] van Duijn CM van Steensel-Moll HA Coebergh JW Risk factors for childhood acute non-lymphocytic leukemia: an association with maternal alcohol consumption during pregnancy? Cancer Epidemiol Biomarkers Prev 1994;3:457–60. 8000294

[17] Farioli A Legittimo P Mattioli S Tobacco smoke and risk of childhood acute lymphoblastic leukemia: findings from the SETIL case-control study. Cancer Causes Control 2014;25:683–92. 24699944 10.1007/s10552-014-0371-9

[18] Mattioli S Farioli A Legittimo P Tobacco smoke and risk of childhood acute non-lymphocytic leukemia: findings from the SETIL study. PLoS One 2014;9:e111028. 25401754 10.1371/journal.pone.0111028 PMC4234298

[19] MacArthur AC McBride ML Spinelli JJ Risk of childhood leukemia associated with parental smoking and alcohol consumption prior to conception and during pregnancy: the cross-Canada childhood leukemia study. Cancer Causes Control 2008;19:283–95. 18283545 10.1007/s10552-007-9091-8

[20] Milne E Greenop KR Scott RJ Parental prenatal smoking and risk of childhood acute lymphoblastic leukemia. Am J Epidemiol 2012;175:43–53. 22143821 10.1093/aje/kwr275

[21] Metayer C Zhang L Wiemels JL Tobacco smoke exposure and the risk of childhood acute lymphoblastic and myeloid leukemias by cytogenetic subtype. Cancer Epidemiol Biomarkers Prev 2013;22:1600–11. 23853208 10.1158/1055-9965.EPI-13-0350 PMC3769478

[22] Schuz J Kaatsch P Kaletsch U Association of childhood cancer with factors related to pregnancy and birth. Int J Epidemiol 1999;28:631–9. 10480689 10.1093/ije/28.4.631

[23] Castro-Jimenez MA Orozco-Vargas LC Parental exposure to carcinogens and risk for childhood acute lymphoblastic leukemia, Colombia, 2000-2005. Prev Chronic Dis 2011;8:A106. 21843409 PMC3181179

[24] Orsi L Rudant J Ajrouche R Parental smoking, maternal alcohol, coffee and tea consumption during pregnancy, and childhood acute leukemia: the ESTELLE study. Cancer Causes Control 2015;26:1003–17. 25956268 10.1007/s10552-015-0593-5

[25] Sorahan T McKinney PA Mann JR Childhood cancer and parental use of tobacco: findings from the inter-regional epidemiological study of childhood cancer (IRESCC). Br J Cancer 2001;84:141–6. 11139329 10.1054/bjoc.2000.1556 PMC2363626

[26] Shu XO Ross JA Pendergrass TW Parental alcohol consumption, cigarette smoking, and risk of infant leukemia: a Childrens Cancer Group study. J Natl Cancer Inst 1996;88:24–31. 8847721 10.1093/jnci/88.1.24

[27] Pang D McNally R Birch JM Parental smoking and childhood cancer: results from the United Kingdom Childhood Cancer Study. Br J Cancer 2003;88:373–81. 12569379 10.1038/sj.bjc.6600774 PMC2747546

[28] Greaves MF Alexander FE An infectious etiology for common acute lymphoblastic leukemia in childhood? Leukemia 1993;7:349–60. 8445941

[29] Brondum J Shu XO Steinbuch M Parental cigarette smoking and the risk of acute leukemia in children. Cancer 1999;85:1380–8. 10189146

[30] Lee KM Ward MH Han S Paternal smoking, genetic polymorphisms in CYP1A1 and childhood leukemia risk. Leukemia Res 2009;33:250–8. 18691756 10.1016/j.leukres.2008.06.031 PMC2787091

[31] Moller H Work in agriculture, childhood residence, nitrate exposure, and testicular cancer risk: a case-control study in Denmark. Cancer Epidemiol Biomarkers Prev 1997;6:141–4. 9037566

[32] Finette BA O’Neill JP Vacek PM Gene mutations with characteristic deletions in cord blood T lymphocytes associated with passive maternal exposure to tobacco smoke. Nat Med 1998;4:1144–51. 9771747 10.1038/2640

[33] Jauniaux E Burton GJ Morphological and biological effects of maternal exposure to tobacco smoke on the feto-placental unit. Early Hum Dev 2007;83:699–706. 17900829 10.1016/j.earlhumdev.2007.07.016

[34] Julvez J Ribas-Fito N Torrent M Maternal smoking habits and cognitive development of children at age 4 years in a population-based birth cohort. Int J Epidemiol 2007;36:825–32. 17550944 10.1093/ije/dym107

[35] Everson RB Randerath E Santella RM Detection of smoking-related covalent DNA adducts in human placenta. Science 1986;231:54–7. 3941892 10.1126/science.3941892

[36] Cumulative Index to IARC monographs on the evaluation of the carcinogenic risk of chemicals to humans. IARC Monogr Eval Carcinog Risk Chem Hum 1986;39:379–403. 3536727

[37] Tredaniel J Boffetta P Little J Exposure to passive smoking during pregnancy and childhood, and cancer risk: the epidemiological evidence. Paediatr Perinat Epidemiol 1994;8:233–55. 7997400 10.1111/j.1365-3016.1994.tb00455.x

[38] Chang JS Parental smoking and childhood leukemia. Meth Mol Biol 2009;472:103–37. 10.1007/978-1-60327-492-0_5 19107431

[39] Boffetta P Tredaniel J Greco A Risk of childhood cancer and adult lung cancer after childhood exposure to passive smoke: a meta-analysis. Environ Health Perspect 2000;108:73–82. 10620527 10.1289/ehp.0010873 PMC1637845

